# Evaluating Workplace Incivility and Its Relationship With Patient Safety Culture Among EMS Staff: A Cross-Sectional Analytical Study in Iran

**DOI:** 10.1155/jonm/8846297

**Published:** 2025-03-25

**Authors:** Amirreza Homaei, Reza Nemati-Vakilabad, Erfan Ebadi, Mirtohid Hosseini, Alireza Mirzaei

**Affiliations:** ^1^Students Research Committee, School of Nursing and Midwifery, Ardabil University of Medical Sciences, Ardabil, Iran; ^2^Department of Medical-Surgical Nursing, School of Nursing and Midwifery, Ardabil University of Medical Sciences, Daneshgah St, Ardabil, Iran; ^3^Department of Critical Care Nursing, School of Nursing and Midwifery, Guilan University of Medical Sciences, Rasht, Iran; ^4^Department of Emergency Nursing, School of Nursing and Midwifery, Ardabil University of Medical Sciences, Ardabil, Iran

**Keywords:** emergency medical services, Iran, patient safety culture, staff, workplace incivility

## Abstract

**Background:** Workplace incivility can severely affect healthcare workers and patients. It creates an unhealthy and unsafe environment, reduces job satisfaction, and often leads to higher staff turnover rates. Therefore, it is crucial to establish a culture of respect and civility in healthcare workplaces to prevent these adverse outcomes.

**Aims:** Research on the relationship between safety culture and workplace incivility in emergency medical service (EMS) settings in Iran needs to be improved, and more studies are required to examine patient safety culture globally. This study examines the relationship between workplace incivility and safety culture among the EMS staff.

**Research Design:** Cross-sectional analytical survey.

**Methods:** This study included 203 EMS staff members who were selected using census population sampling from emergency medical centers in Ardabil City. Researchers used the EMS–Safety Attitudes Questionnaire (EMS–SAQ), the workplace incivility scale, and a demographic characteristics' form to gather information. The study employed multivariable logistic regression and multiple linear regression analyses to analyze the impact of various factors on workplace incivility and safety culture amongst EMS staff.

**Results:** According to the study, EMS workers' average patient safety culture was 51.32%. The study also revealed that more than half of EMS workers (52.7%) experience workplace incivility at least once a month. This behavior negatively affects the staff's adherence to patient safety guidelines during EMS missions, leading to high turnover rates. The study also found that workplace incivility is linked to patient safety culture, and these negative experiences can decrease patient safety culture.

**Conclusion:** EMS workers in Iran exhibit a poor attitude toward patient safety culture, exacerbated by workplace incivility. This negative behavior impacts adherence to safety guidelines and contributes to high staff turnover intention rates. To improve outcomes, healthcare organizations need to implement policies and training programs to address inappropriate behaviors. Cultivating a culture of respect, professionalism, and effective communication can enhance staff safety and improve the quality of patient care.

**Summary**

• **Implications for nursing management:** EMS staff face incivility at work, which harms patient safety and staff's well-being. To address this, managers should implement policies and training programs prioritizing patient safety and protocol adherence, conflict resolution, and communication skills. Policymakers should encourage a culture of respect, establish support systems, and advocate for policies prioritizing staff well-being and patient safety.
• **Patient or public contribution:** No patient or public contribution.

## 1. Introduction

Medical emergencies are sudden and unexpected medical events that require immediate treatment. In general, urgency refers to a situation in which the patient's life is threatened immediately or within a few minutes to a few hours [1]. These events usually lead to the death of the patient in the absence of quick medical intervention, such as severe trauma from car accidents, heart attacks, strokes, emergency childbirth, and acute abdomen issues. In these cases, the emergency medical center usually intervenes [2]. In Iran, paramedics hold nursing, anesthesia, and emergency medicine degrees. They provide specialized medical services outside of hospitals. To qualify, individuals must complete an accredited training program and obtain certification in emergency medical services (EMSs) [3].

Emergency medical technicians (EMTs) face certain dangerous elements and complex problems in care situations, including unpredictable challenges and sudden changes in care decisions. They also encounter numerous ethical issues [4]. Consequently, decision-making can be complex and challenging, often creating mental pressure on technicians [5]. Safety culture refers to workers' collective beliefs and perceptions regarding the organization and safety of their workplace operations [6]. Recent research has examined safety culture in various healthcare settings, including hospital inpatient units, intensive care units (ICUs), nursing wards, ambulatory care, and skilled nursing facilities [7, 8]. However, only some studies have evaluated workplace safety culture, specifically in EMS [6].

EMS providers serve patients in critical and complex conditions, exposing them to unpredictable stressors and threats [5]. As a result, they experience high levels of stress, leading to chronic stress–related complications. Studies indicate that approximately 22% of EMS providers deal with stress-related issues, such as anxiety, irritability, social isolation, sleep disorders, job dissatisfaction, burnout, workplace incivility, leaving the profession, suicide, posttraumatic stress disorder, risk behaviors, psychological problems, and depression; they are also more likely to commit medical errors [9–11]. Various factors influence the attitudes of EMS personnel toward their organization and work environment, including beliefs, experiences, and personality traits. Personnel experiences, shaped by interactions with patients, colleagues, and system managers, play a crucial role in this context [12]. Among these interactions, disrespect and incivility significantly influence personnel attitudes toward their organization. Incivility is negative interpersonal actions that violate social interaction norms, ranging from simple etiquette violations to outright harassment [13, 14].

One factor potentially associated with incivility in EMS is the organizational culture of the workplace. Previous studies have shown that nurses' experiences with disrespectful behaviors have diminished their patient safety, particularly regarding cooperation with healthcare colleagues and recognizing patient safety–related factors [7]. Furthermore, experiences of public disrespect have negatively impacted nurses' communication abilities, risk management related to safety issues, and overall safety. Incivility in the workplace adversely affects the quality of professional life and the performance of nurses in providing care, ultimately reducing the quality of nursing care [15].

While previous studies have explored workplace incivility, a notable gap remains in the literature regarding its effects on EMS personnel's perceptions of patient safety culture. Understanding this relationship is crucial for enhancing workplace dynamics and improving patient outcomes. Patient safety culture encompasses the shared beliefs, values, and attitudes guiding healthcare workers' commitment to patient safety. This culture plays a vital role in effective healthcare delivery, influencing how employees address safety concerns, report errors, and collaborate.

Our research addresses this gap by examining the relationship between patient safety culture and workplace incivility among EMS personnel based in Ardabil, a city and the capital of Ardabil Province in Iran. Specifically, we will assess the frequency of workplace incivility experienced by EMS staff affiliated with the University of Medical Sciences in Ardabil and their perceptions of patient safety culture. We aim to identify any potential correlations between these two factors. We anticipate that our findings will yield valuable insights into the impact of workplace incivility on patient safety culture. These insights will inform interventions designed to reduce incivility and foster a more favorable safety culture within the EMS environment, ultimately contributing to a safer and more respectful workplace.

## 2. Methods

### 2.1. Design and Participants

This cross-sectional analytical study focused on EMS staff working in emergency medical centers in Ardabil City, which has a population of approximately 1,270,420 and is home to five hospitals. The study encompassed EMS personnel from 13 urban and 14 nonurban bases in the surrounding areas. Each base generally employs seven to eight personnel who work 12- or 24-h shifts. In the emergency medical bases in Iran, each team typically consists of two personnel working 24-h shifts, with their primary responsibility being transporting patients to hospitals during emergencies. During periods without urgent missions, these personnel have the opportunity to rest, helping them manage fatigue and work pressure effectively. This structure enables them to provide services with increased energy and readiness. Participants were required to have at least 6 months of work experience and a willingness to join the study. Employees located at headquarters were excluded from the study due to substantial differences in their roles compared to field staff, which could potentially skew the results. In addition, individuals who had not completed the necessary questionnaires were excluded to ensure the integrity of the data collected.

An a priori power analysis was conducted using G⁣^∗^ Power 3.1 to ascertain the study's sample size. With 18 predictor variables included in the regression model, the analysis suggested a sample size of 134 participants, assuming a medium effect size, a significance level of 0.05, and a statistical power of 0.80. The study population comprised 232 individuals, of which, 212 were deemed eligible, including 198 EMS personnel and 14 dispatch personnel. Researchers utilized census population sampling to compile a comprehensive list of personnel from urban and roadside EMS stations, ensuring the inclusion of all pertinent individuals. Two hundred and twelve questionnaires were distributed in total. However, nine responses were excluded due to incomplete information, resulting in a final sample size of 203 EMS personnel working in emergency medical centers in Ardabil City, which yielded a response rate of approximately 95%.

Participants were informed of their right to withdraw from the study without facing any repercussions. To protect the anonymity of each participant, we assigned a distinct identifier code to every individual. This code was printed on their questionnaire, ensuring their responses remained confidential and could not be traced back to them. This approach allowed us to collect valuable data while safeguarding the privacy of all participants involved in the study. This code served as their identifier for the entire duration of the study. If participants chose to withdraw, they could notify the research team by emailing a designated address and their unique identifier code. This procedure ensured the confidentiality of their identities, as we would only use the code to remove their data without accessing their names. Questionnaires were distributed in person, and participants were made aware of the withdrawal process through the information provided on the front page of the questionnaire. This approach promoted a transparent and confidential method for participants to withdraw their data. To encourage participation in the study, a reward of a 2,000,000 rials' gift card or a complimentary book was offered to participants through a lottery system upon completion of the survey. Following ethical guidelines and obtaining the necessary approvals, the research study was authorized by the Ardabil University of Medical Sciences, referenced as IR.ARUMS.REC.1402.060. Two hundred and twelve questionnaires, including the EMS–Safety Attitudes Questionnaire (EMS–SAQ) and the Workplace Incivility Scale (WIS), were distributed. Data collection occurred from July 1 to September 1, 2023.

### 2.2. Data Collection

#### 2.2.1. Demographic Characteristics' Form

The demographic characteristics' form included various factors such as age, work experience, gender, marital status, educational status, workplace, type of employment, annual income (USD), kind of shift, job position, overtime, number of night shifts, satisfaction with economic status (rated from weak = 1 to excellent = 5), satisfaction with social status (rated from poor = 1 to excellent = 5), turnover intention (with most satisfied/least intent to quit = 1 and most dissatisfied/most intent to quit = 5), and an assessment of compliance with patient safety during missions (rated from poor = 1 to excellent = 5).

#### 2.2.2. WIS

Workplace incivility was measured using the modified WIS [14], which includes two subgroups of immediate supervisors and coworkers. In this study, we used the modified 20-item version of this questionnaire adapted by Cash et al., of which, 10 items were related to immediate supervisors and 10 were related to coworkers [16]. Typically, the WIS is based on incivility experience from rarely (once every few months or less), rarely (about once a month), and sometimes (at least once a week) to too often (at least once a day). For this evaluation, consistent with previous research, it was recognized that workplace incivility may be a common occurrence in EMS settings. Thus, respondents were classified as experiencing regular incivility if they reported that any of the listed behaviors occurred once a week or more during the past 12 months [16]. In Cortina et al.'s study, Cronbach's alpha for this questionnaire was reported as 0.89 [14]. In this study, Cronbach's alpha for rudeness experienced by the supervisor was 0.80 and that of the coworkers was 0.87.

### 2.3. EMS–SAQ

Peterson et al. developed the original 60-item EMS–Safety Attitudes Questionnaire (EMS–SAQ) [6]. Subsequently, Venesoja et al. pioneered the application of a 30-item instrument designed to evaluate patient safety culture in Finnish emergency medicine [17]. Our study assessed patient safety culture in EMSs using a psychometrically validated 30-item version of the EMS–SAQ in Persian, evaluated in 2024 by Norouzinia et al. [18]. Factor analysis indicated that this shorter version effectively assesses six key areas of workplace safety culture: safety atmosphere, teamwork atmosphere, management perception, job satisfaction, working conditions, and stress detection [17]. The EMS–SAQ collects respondents' perceptions of various safety culture aspects, including management attitudes and teamwork climate. Responses are scored on a five-point Likert scale, ranging from 0 (*strongly disagre*e) to 100 (*strongly agree*) [6]. Standard scoring involves calculating the mean domain score and the percentage of positive responses [6]. According to a study by Patterson et al. and Venesoja et al., two questions were reverse-coded to maintain consistency: SC4, “In this EMS agency, it is challenging to discuss errors,” and TWC2, “At this EMS agency, it is difficult to voice concerns about patient care issues” [6, 17]. The reported Cronbach's alpha for the subgroups in our study ranged from 0.68 to 0.84, reflecting good internal consistency. In Venesoja et al.'s study, Cronbach's alpha for the subgroups was reported between 0.70 and 0.73 [17]. Safety culture domain scores were categorized into “strong” (≥ 75) and “weak” (< 75), with participants needing to answer “slightly agree” or higher to indicate a strong perception of safety culture.

The WIS underwent a comprehensive translation process into Persian, which commenced with obtaining written authorization from the instrument's manufacturer. Two proficient bilingual individuals conducted the initial translation and subsequently performed a back-translation to ensure fidelity to the original instrument. Following this, two subject matter experts rigorously reviewed the back-translated version to assess its accuracy and contextual relevance. A specialist in Persian language and literature further refined the translation to enhance linguistic clarity and cultural appropriateness. A panel of twenty experts evaluated the items and provided valuable feedback to establish the questionnaire's content validity, resulting in necessary modifications based on their insights.

The content validity index (CVI) was calculated for each item, yielding a CVI of 0.93 for the EMS–SAQ and 0.94 for the WIS, indicating a strong content validity level [19]. Furthermore, the content validity ratio (CVR) was calculated using the assessments of twelve experts, producing a CVR of 0.94 for the WIS and 0.91 for the EMS–SAQ. These high CVR values further affirm the validity of the questionnaires, reinforcing their appropriateness for the target population [20].

### 2.4. Data Analysis

The collected data were analyzed using the robust SPSS 22v statistical software. Descriptive statistics, including frequency counts, percentages, means, and standard deviations, summarized the data effectively. The dependent variable for this analysis was workplace incivility. In contrast, the independent variables included satisfaction with economic status, satisfaction with social situations, turnover intention, and compliance with patient safety in EMS missions.

Before conducting the multivariable logistic regression, the raw data underwent several transformations. Categorical variables were coded appropriately, and continuous variables were normalized when necessary to ensure they met the assumptions required for the analysis. Following this preparation, Pearson correlation analysis was utilized to explore relationships among the variables. We hypothesized a negative correlation, suggesting that poorer workplace incivility scores (indicating higher levels of incivility) are associated with lower EMS–SAQ scores (indicating poorer safety attitudes). Adjusted binary logistic regression and a 95% confidence interval were employed to assess the impact of independent variables on the likelihood of experiencing workplace incivility. In addition, collinearity statistics, specifically tolerance and variance inflation factor (VIF), were calculated to evaluate potential multicollinearity among the independent variables, ensuring that the assumptions of the regression analyses were satisfied.

Furthermore, a multiple linear regression analysis was performed, with the dependent variable being patient safety culture. The independent variables included dimensions of workplace incivility and various demographic factors. This analysis aimed to determine how these factors influenced perceptions of patient safety culture among EMS staff. The model selection process was guided by significance levels and theoretical relevance, determining which variables were included in the regression models. The rationale behind the chosen statistical tests was discussed, emphasizing their appropriateness for the research questions and the underlying assumptions necessary for valid interpretations of the results.

### 2.5. Ethics Approval and Consent to Participate

The Ethics Committee of the Ardabil University of Medical Sciences approved the research study with the reference number: IR.ARUMS.REC.1402.060. Before participating in the study, all individuals were provided with detailed information about the research and its significance, and they gave their informed written consent. The participants completed the questionnaire anonymously. Furthermore, all procedures followed in the study were by the 1964 Declaration of Helsinki, which sets forth ethical principles for medical research involving human subjects.

## 3. Results

The demographic characteristics of the sample community are summarized in Table 1. Participants had a mean age of 30.71 years and an average work experience of 7.25 years. A significant majority identified as male, while a small percentage were female. The marital status was nearly evenly split between single and married individuals. In terms of education, most participants held a bachelor's degree, followed by those with an associate degree and a few with advanced degrees. The majority were employed in urban settings, specifically Ardebil City. Employment types varied, including commitment, contractual, and employed statuses. Most participants reported an annual income within the $2800–3600 range and primarily worked 12-h shifts. The data also indicated variability in overtime, night shifts, turnover intention, satisfaction with economic status, and patient safety compliance.

Table 2 presents the descriptive statistics for the study variables based on a sample of 203 participants. The scores reflect the aggregated dimensions derived from individual survey items, which were averaged to compute the overall scores for each construct. Participants reported a moderate teamwork climate (*M* = 50.12, SD = 16.91), highlighting a balanced perception of management (*M* = 46.58, SD = 17.77) and stress recognition (*M* = 58.43, SD = 22.67). In addition, work conditions (*M* = 49.53, SD = 19.90) and safety climate (*M* = 48.48, SD = 16.94) ratings were also moderate. Job satisfaction (*M* = 54.77, SD = 22.95) and overall patient safety culture (*M* = 51.32, SD = 12.93) displayed average levels. However, participants reported experiences of workplace incivility, particularly from immediate supervisors (*M* = 1.46, SD = 0.49) and coworkers (*M* = 1.23, SD = 0.42), indicating ongoing challenges in the work environment.

Table 3 details the correlations among the study variables, calculated from the identical aggregated scores for each dimension. Strong positive correlations were identified between the teamwork climate and safety climate (*r* = 0.729, *p* < 0.01), as well as between job satisfaction and patient safety culture (*r* = 0.787, *p* < 0.01). Conversely, experiences of workplace incivility from immediate supervisors were significantly negatively correlated with several factors, including teamwork climate (*r* = −0.380, *p* < 0.01) and job satisfaction (*r* = −0.349, *p* < 0.01), suggesting that such incivility has detrimental effects on overall workplace dynamics.

The multivariable logistic regression model examined the relationship between workplace incivility (the dependent variable) and various independent variables, including economic satisfaction, social satisfaction, turnover intention, and compliance with patient safety protocols during EMS missions, as presented in Table 4. The findings revealed that frequent incivility from immediate supervisors significantly impacted turnover intention (OR = 0.693, *p*=0.004) and adherence to patient safety protocols (OR = 0.741, *p*=0.018). Furthermore, concerning coworker incivility, turnover intention was also significantly affected (OR = 0.689, *p*=0.012), while compliance with patient safety was not statistically significant (OR = 0.941, *p*=0.672).

Table 5 outlines the impact of workplace incivility on patient safety culture among EMS staff, highlighting incivility from supervisors and coworkers as a significant predictor. In this multiple linear regression analysis, the dependent variable is patient safety culture, while the independent variables comprise supervisor incivility, coworker incivility, and levels of economic and social satisfaction. The findings indicate that supervisor incivility exerts a strong negative effect on patient safety culture (*B* = −7.267, *p* < 0.001), with coworker incivility also demonstrating a significant adverse impact (*B* = −3.958, *p*=0.040). Furthermore, economic and social satisfaction positively influenced patient safety culture, albeit to a lesser extent.

## 4. Discussion

This study explores the crucial connection between workplace behavior and the safety culture in EMSs. Negative interactions in the workplace can have a detrimental effect on team dynamics, resulting in decreased job satisfaction and increased turnover rates, which can compromise patient safety. By examining these aspects, the study seeks to provide valuable insights that can help develop strategies to promote an atmosphere of respect and safety in prehospital emergency environments. This approach is expected to enhance employee well-being and patient care quality.

The study's results determined that the overall average patient safety culture in EMS workers was 51.32%, which shows that Iranian EMS staff had a weak attitude toward patient safety culture, according to previous studies [6, 17]. In a survey by Kosydar-Bochenek et al., EMS workers demonstrated a more favorable attitude toward patient safety culture than their Iranian counterparts [21]. EMS workers are exposed to various risks in their workplace, which makes workplace safety culture extremely diverse. To improve the safety culture in this setting, it is essential to conduct regular training sessions, create an environment where staff can report incidents accurately, promote healthy collaboration and communication, periodically assess patient safety culture, and reward safe behaviors. EMS staff have a relatively better attitude toward patient safety culture. However, Iranian EMS staff should enhance their safety culture. The diverse nature of workplace safety culture is not surprising, given the many risks the EMS work environment poses to patients and providers. By organizing regular training sessions, creating an environment where staff can report incidents accurately, promoting healthy collaboration and communication, periodically assessing patient safety culture, and rewarding safe behaviors, we can achieve the objective of improving workplace safety culture in EMS settings.

The study identified that stress recognition scored highest (58.43%) and perception of management scored lowest (46.58%). Different subgroups of safety culture showed significant differences in scores. Other studies found varying scores, with stress recognition and job satisfaction scoring the highest and lowest, respectively, and job satisfaction and working environment conditions are two important factors. EMS work environment poses many risks to patient and provider safety, with varying factors underlying cultural differences such as regional practices, economic resources, and leadership structures and styles. No international standard exists for classifying and reporting adverse events, medical malpractice, and errors in EMS [6]. Stress recognition in EMS staff refers to the capacity to identify and acknowledge stress among EMS staff. This includes being aware of the signs and symptoms of stress in oneself and coworkers and taking effective steps to address and manage it to maintain well-being [6]. EMS providers experience occupational stressors that negatively impact their health and job performance [9]. One effective approach to reducing stress is recognizing and managing it effectively [22]. Emergency personnel's perceptions of management reflect the organization's management style, behavior, and social skills [21]. A safety culture is shaped by its leaders, managers, employees, and supervisors. Leadership is vital in promoting teamwork and safety; management safety commitment in healthcare is linked to collaboration. The safety culture has a significant impact on system safety [23]. In the last 10 years, many EMS organizations have taken the lead in changing how EMS leaders and physicians view errors and their responsibility to prevent them. Establishing a safety culture and implementing a safety management system are critical objectives for organizations that aspire to be safe and highly reliable [24]. To enhance the management and diagnosis of stress and to promote effective leadership in EMS staff, it is essential to conduct regular training courses and workshops. These programs should focus on stress diagnosis and management, awareness, and effective strategies for handling stress in challenging situations. In addition, to improve the perception of leadership, it is necessary to provide constructive feedback, create an open space for communication, build organizational trust, encourage cooperation and interaction with the team, and promote effective leadership concepts. By implementing these measures, we can significantly improve the perception of leadership and organizational culture and enhance the overall performance of the EMS staff.

Our study found that over half of EMS workers (52.7%) experience workplace incivility at least once a month. However, Cash et al. reported a prevalence of 71.2% [16], which is higher than this study's results, but Liu et al. found a lower prevalence of 32.1% [25]. It is a common issue in healthcare workplaces, with up to 90% of healthcare workers experiencing it [26, 27]. Alquwez and Alshehry et al.'s studies indicate that about two-thirds of the nurses surveyed understood rudeness in the workplace and its dimensions [28, 29]. This can be attributed to Iran's complex and high-pressure environment of EMS centers, which can lead to workplace incivility behavior. The lack of strict laws and disciplinary systems to address such behavior also contributes to it. The researchers suggest that EMS managers should take workplace incivility seriously and work to eliminate it to prevent its negative impact on staff's caring behavior and work outcomes.

The multivariable logistic regression analysis revealed that workplace incivility from immediate supervisors and coworkers significantly affects turnover intention among EMS staff, aligning with findings from previous studies in various contexts [16, 30–32]. Research indicates that when employees encounter disrespectful behavior from supervisors or coworkers, it often leads to dissatisfaction, demotivation, and an increased desire to leave the organization [16, 33]. Such negative interactions can erode trust, damage professional relationships, and foster a toxic work environment, ultimately heightening turnover intentions [32]. This phenomenon is not unique to EMS personnel; similar trends have been observed in other healthcare settings, suggesting that preventing workplace incivility is crucial for maintaining a positive work culture and reducing turnover across diverse environments. One practical recommendation is implementing training programs promoting respectful communication and behavior. Encouraging open dialog, empathy, and mutual respect can help mitigate incidents of incivility and enhance overall employee satisfaction. Establishing regular feedback mechanisms and clear channels for reporting uncivil behavior is essential for promptly addressing issues and fostering a respectful workplace.

Our study also found that workplace incivility from immediate supervisors directly impacts compliance with patient safety during EMS missions. This finding echoes the Alquwez study, which demonstrated that public rudeness negatively affects patient safety competence and impairs nurses' ability to ensure safe care [7, 34, 35]. The implications of workplace incivility extend beyond individual experiences; they can create systemic issues that compromise patient safety [36]. In different cultural contexts, such as those examined in Aljuaid et al.'s research, a moderate and significant negative relationship between incivility and patient safety was also observed [37]. These studies collectively underscore the necessity of establishing policies that address uncivil behavior in healthcare settings while enhancing the competency of healthcare professionals in patient safety. Workplace incivility's physical and emotional impacts can harm the overall work environment and lead to unsafe patient care practices [36]. Therefore, healthcare organizations must provide comprehensive policies and training to identify, prevent, and manage incivility. This process fosters respect and professionalism, enhances comprehension of effective communication strategies, and advances conflict resolution competencies.

Our study found a strong association between workplace incivility from supervisors or coworkers and a negative safety culture among EMS staff, consistent with findings from other studies across different healthcare systems [28]. Workplace incivility, characterized by rude, disrespectful, or insensitive behavior, can manifest in various forms, such as ignoring colleagues, making derogatory remarks, spreading rumors, and other forms of verbal aggression [16]. The negative impact of incivility on patient safety has been highlighted in diverse healthcare settings, with Alquwez's study indicating that uncivil behavior from coworkers and the public detrimentally affects nurses' patient safety competence [7]. It emphasizes the urgent need for policies that promote respect and professionalism in healthcare settings to improve patient outcomes and satisfaction. The findings from the survey conducted by Schoville et al. further corroborate this perspective, demonstrating that incivility has a detrimental impact on patient safety and overall outcomes [38]. It is essential to acknowledge that participants experiencing a poor teamwork climate, negative perceptions of management, stress recognition, unfavorable work conditions, and low job satisfaction may report higher incivility. Therefore, addressing workplace incivility is crucial for fostering a positive safety culture among emergency service workers. Implementing policies that promote respect and professionalism can significantly enhance patient outcomes, as impoliteness in the workplace diminishes healthcare professionals' ability to ensure patient safety and negatively affects their clinical performance.

Our study found that EMS staff with higher education levels and commitment to their employment rated the safety culture lower than their colleagues with lower education levels and employment status. This finding is consistent with previous research [17]. However, the results obtained were inconsistent with the findings of earlier studies [6, 28]. EMS staff with higher education levels and those in more stable positions tend to have fewer job contacts. Thus, this finding implies that the lack of social interaction can hurt the safety climate and behavior within EMS organizations. Initially, newly graduated nurses enter a commitment status for 2 years after graduation, during which they are required to gain practical experience in a clinical setting. Following this commitment period, they must take a national employment exam administered by the National Organization for Educational Testing and the Ministry of Health. Upon passing this exam, their employment status changes to contractual, offering them better pay and benefits than during the commitment phase. After accumulating 8 years of clinical experience and acquiring the necessary skills and qualifications, nurses can transition to an employed status, a formal employment position with even higher salaries and additional benefits. While all three statuses are considered employed, the key differences lie in the duration of service, pay, and benefits. This structured pathway allows nurses to advance in their careers, gaining valuable experience gradually. Given these dynamics, EMS agencies need to be aware of social interaction and take steps to ensure that all personnel, regardless of their education level or employment status, have access to opportunities for social interaction and ongoing professional development. By promoting a positive safety culture and providing opportunities for personal and professional growth, EMS agencies can help mitigate the adverse effects of limited job contacts and ensure that all personnel can perform their duties safely, effectively, and confidently.

The study's findings showed that EMS staff satisfaction with their social situations predicts patient safety culture. Social satisfaction in nursing is a complex and multidimensional idea that refers to how contented individuals are with the way they interact socially with healthcare providers [39]. People from higher social classes prioritize their health and safety by being more aware of health and wellness concerns. However, people with lower socioeconomic status may value their health and safety less [40]. Social satisfaction covers a range of factors, including the quality of care provided, the level of respect and empathy shown by nurses, the degree of personalization in the care plan, and the extent to which patients are empowered to participate in their care. The social satisfaction of patients and the job satisfaction of nurses are influenced by various factors such as cultural norms, healthcare policies, and resource availability, which vary based on the context of each society and healthcare system.

Our research reveals that following patient safety guidelines during EMS missions significantly predicts safety culture. Prehospital care is provided outside medical facilities in a constantly changing and challenging environment. Caregivers evaluate patients with varying symptoms and conditions, sometimes highly severe [41]. They use different types of vehicles and equipment unrelated to the hospital environment. Care is provided in an unstable environment due to uncontrolled patient volume, variable levels of consciousness, and lack of information, among other factors [42]. EMS providers who prioritize patient safety and adhere to established protocols work in a safety-focused environment that values safety and takes proactive measures to mitigate risks [21, 43]. Adhering to safety standards can enhance patients' outcomes and improve healthcare practitioners' efficiency and effectiveness [44]. Therefore, prioritizing patient safety during EMS missions is crucial for promoting a safety culture within the organization.

## 5. Limitations

This innovative research study in Iran examines the significant relationship between workplace incivility and safety culture among EMS personnel. The large sample size enhances the reliability of the findings; however, certain limitations may affect the generalizability of the results. Notably, the gender imbalance, comprising only seven female respondents out of 203, raises concerns about potential male dominance in the outcomes, which may not accurately reflect the broader EMS workforce in Iran. This finding is consistent with previous studies [45, 46], indicating a predominance of male participants, as men primarily occupy roles in the prehospital emergency department. At the same time, women are mainly employed in the dispatch department.

Cultural differences and varying socioeconomic conditions may also limit the study's findings' applicability. The cross-sectional design captures a snapshot of workplace dynamics but restricts causal inferences. In addition, self-reporting questionnaires could introduce response bias, as participants may provide socially desirable answers. The study highlights that greater incivility may be linked to poor teamwork climates, negative management perceptions, and low job satisfaction. However, the exclusion of administrative staff may overlook critical workplace dynamics. Future research should consider random sampling, ensure confidentiality, and address potential biases while recognizing cultural contexts. Although the sample size of 212 is robust, it may still fall short of recommended thresholds, and the historically low female participation in EMS is a concern. Future studies should aim for balanced gender representation, refine inclusion criteria, and explore additional predictor variables. Longitudinal studies would be beneficial for observing changes over time and improving generalizability across diverse EMS settings.

## 6. Conclusion

Our study raises significant concerns regarding the attitudes of EMS personnel in Iran towards patient safety culture, as evidenced by an average score of just 51.32%. Disturbingly, more than half of the EMS workforce (52.7%) reported experiencing workplace incivility at least once a month, negatively impacting their adherence to patient safety protocols during emergency missions and contributing to high turnover rates. Furthermore, the findings demonstrate a connection between workplace incivility and the safety culture within EMS settings, indicating that adverse experiences can undermine the culture of safety. To improve patient outcomes and satisfaction, healthcare organizations must implement comprehensive policies and training programs to identify, prevent, and manage inappropriate behaviors. By fostering a culture of respect, professionalism, and effective communication, EMS agencies can cultivate a safer and more supportive work environment for their staff. In addition, enhancing conflict resolution skills will be vital in promoting a healthy workplace where employees feel valued, respected, and secure. These initiatives are essential for advancing the quality of patient care and ensuring a positive organizational culture.

## 7. Implications for Nursing Management

EMS staff often face incivility in the workplace, which can negatively impact patient safety and staff well-being. To address this issue, EMS managers can implement policies and training programs prioritizing patient safety and protocol adherence. Prioritizing training programs that focus on conflict resolution and communication skills is crucial. Policymakers can promote a culture of respect and civility in the workplace. Support systems should be established to help EMS staff cope with workplace challenges. Regular research and evaluation are necessary to monitor workplace dynamics. Collaboration and advocacy efforts must advocate for policies prioritizing staff well-being and patient safety in healthcare settings.

## Figures and Tables

**Table 1:** Characteristics of participants (*n* = 203).

**Characteristics**		**Mean**	**SD**

Age		30.71	6.473

Work experience		7.25	5.776

		***n***	**%**

Gender	Male	196	96.6
	Female	7	3.4

Marital status	Single	105	51.7
	Married	98	48.3

Educational status	Associate degree	72	35.5
	Bachelor's degree	117	57.6
	Master's or Ph.D.	14	6.9

Workplace	Ardebil City	162	79.8
	Countryside	41	20.2

Type of employment	Commitment	70	34.5
	Contractual	54	26.6
	Employed	79	38.9

Annual income (US$)	1600–2200	22	10.8
	2200–2800	44	21.7
	2800–3600	87	42.9
	> 3600	50	24.6

Type of shift	12 h	114	56.2
	24 h	89	43.8

Position	EMT–intermediate	141	69.5
	EMT–paramedic	62	30.5

Overtime	≤ 40	32	15.8
	41–80	109	53.7
	81–120	34	16.7
	> 120	28	13.8

Number of night shift	0–3	10	4.9
	4–7	89	43.8
	8–11	77	37.9
	> 12	27	13.3

Satisfaction with economic status	Completely dissatisfied	135	66.5
	Little dissatisfied	37	18.2
	No idea	20	9.9
	Little satisfied	9	4.4
	Completely satisfied	2	1.0

Satisfaction with the social situation	Completely dissatisfied	81	39.9
	Little dissatisfied	60	29.6
	No idea	24	11.8
	Little satisfied	32	15.8
	Completely satisfied	6	3.0

Turnover intention	Most satisfied/least intended to leave	16	7.9
	Little dissatisfied	69	34.0
	No idea	21	10.3
	Little satisfied	72	35.5
	Most dissatisfied/most intended to leave	25	12.3

How do you evaluate the level of compliance with patient safety in your missions?	Weak	65	32.0
	Medium	48	23.6
	No idea	23	11.3
	Good	46	22.7
	Excellent	21	10.3

**Table 2:** Descriptive statistics of the study variables (*n* = 203).

Variable	Mean	SD	Min	Max
Teamwork climate	50.12^b^	16.91	8.33	91.67
Perception of management	46.58^b^	17.77	6.25	87.50
Stress recognition	58.43^b^	22.67	0.00	100.00
Work conditions	49.53^b^	19.90	6.25	93.75
Safety climate	48.48^b^	16.94	3.57	85.71
Job satisfaction	54.77^b^	22.95	0.00	100.00
Patient safety culture total	51.32^b^	12.93	18.01	81.32
Immediate supervisor's workplace incivility	1.46	0.49	1.00	2.00
Coworker workplace incivility	1.23	0.42	1.00	2.00

^a^Strengths.

^b^Weaknesses.

**Table 3:** Correlations among the study variables (*n* = 203).

Variables	1	2	3	4	5	6	7	8	9
Teamwork climate	1	—	—	—	—	—	—	—	—
Perception of management	0.601⁣^∗∗^	2	—	—	—	—	—	—	—
Stress recognition	−0.140⁣^∗^	−0.002	1	—	—	—	—	—	—
Work conditions	0.596⁣^∗∗^	0.481⁣^∗∗^	−0.113	1	—	—	—	—	—
Safety climate	0.729⁣^∗∗^	0.590⁣^∗∗^	−0.251⁣^∗∗^	0.697⁣^∗∗^	1	—	—	—	—
Job satisfaction	0.642⁣^∗∗^	0.520⁣^∗∗^	−0.091	0.486⁣^∗∗^	0.615⁣^∗∗^	1	—	—	—
Patient safety culture total	0.817⁣^∗∗^	0.766⁣^∗∗^	0.151⁣^∗^	0.760⁣^∗∗^	0.800⁣^∗∗^	0.787⁣^∗∗^	1	—	—
Immediate supervisor's workplace incivility	−0.380⁣^∗∗^	−0.291⁣^∗∗^	0.047	−0.215⁣^∗∗^	−0.278⁣^∗∗^	−0.349⁣^∗∗^	−0.355⁣^∗∗^	1	—
Coworker workplace incivility	−0.196⁣^∗∗^	−0.138⁣^∗^	0.078	−0.100	−0.168⁣^∗^	−0.136	−0.154⁣^∗^	0.297⁣^∗∗^	1

*Note:* 1, Teamwork climate; 2, perception of management; 3, stress recognition; 4, work conditions; 5, safety climate; 6, job satisfaction; 7, patient safety culture total; 8, immediate supervisor's workplace incivility; 9, coworker workplace incivility. All variable correlations were significant at ⁣^∗^*p* < 0.01 and ⁣^∗∗^*p* < 0.001.

**Table 4:** The multivariable logistic regression model of factors related to those experiencing frequent once a week or more incivility, from each source (*n* = 203).

	Variables	*p* value	OR	95% CI	
				Lower	Upper
Immediate supervisor's workplace incivility	Satisfaction with economic status	0.708	1.073	0.741	1.553
	Satisfaction with the social situations	0.871	0.975	0.717	1.326
	Turnover intention	0.004	0.693	0.541	0.889
	The level of compliance with patient safety in EMS missions	0.018	0.741	0.577	0.950

Coworker workplace incivility	Satisfaction with economic status	0.383	1.200	0.797	1.809
	Satisfaction with the social situations	0.655	1.082	0.766	1.530
	Turnover intention	0.012	0.689	0.516	0.920
	The level of compliance with patient safety in EMS missions	0.672	0.941	0.708	1.249

**Table 5:** Results of the multiple regression analysis on the EMS staff's patient safety culture (*n* = 203).

Variables	*B*	SE-b	*t*	*p* value	95% confidence interval		Collinearity statistics	
					Lower	Upper	Tolerance	VIF
Constant	63.668	9.126	6.976	*p* < 0.001	45.664	81.672		
Immediate supervisor's workplace incivility	−7.267	1.730	−4.200	*p* < 0.001	−10.680	−3.854	0.759	1.317
Coworker workplace incivility	−3.958	1.917	−2.065	0.040	−7.741	−0.176	0.852	1.174
Age	−0.352	0.203	−1.731	0.085	−0.753	0.049	0.328	3.048
Gender	4.937	4.490	1.099	0.273	−3.922	13.795	0.842	1.188
Marital status	0.050	2.093	0.024	0.981	−4.079	4.179	0.517	1.936
Educational level	−3.456	1.422	−2.430	0.016	−6.262	−0.651	0.817	1.224
Workplace	−1.057	2.165	−0.488	0.626	−5.328	3.215	0.748	1.337
Type of employment	−2.578	1.333	−1.935	0.049	−5.207	0.051	0.435	2.301
Annual income (US$)	1.830	1.075	1.702	0.090	−0.291	3.951	0.567	1.763
Type of shift	2.695	1.796	1.501	0.135	−0.847	6.238	0.712	1.405
Position	3.137	1.908	1.644	0.102	−0.627	6.901	0.732	1.367
Overtime	0.199	1.085	0.184	0.855	−1.941	2.340	0.604	1.657
Number of night shift	−1.259	1.141	−1.104	0.271	−3.510	0.991	0.717	1.394
Satisfaction with economic status	1.882	1.028	1.830	0.069	−0.146	3.910	0.645	1.550
Satisfaction with the social situations	1.832	0.816	2.245	0.026	0.222	3.442	0.607	1.649
The level of compliance with patient safety in EMS missions	1.627	0.678	2.399	0.017	0.289	2.965	0.626	1.597

*Note:R* ^2^, 0.37; Adjusted *R*^2^, 0.31; F (6.780), Durbin–Watson, 1.66; *p* < 0.001; A Dependent variable: Patient safety culture.

## Data Availability

Data are available from the corresponding author (Alireza Mirzaei) upon request.
